# A pictorial presentation of 3.0 Chicago Classification for esophageal motility disorders

**DOI:** 10.1590/S1679-45082016MD3444

**Published:** 2016

**Authors:** Fernando Augusto Herbella, Priscila Rodrigues Armijo, Marco Giuseppe Patti

**Affiliations:** 1 Escola Paulista de Medicina, Universidade Federal de São Paulo, São Paulo, SP, Brazil; 2 University of Chicago, Chicago, Illinois, Estados Unidos

**Keywords:** Manometry/methods, Esophageal motility disorders, Esophageal achalasia/classification

## Abstract

High resolution manometry changed several esophageal motility paradigms. The 3.0 Chicago Classification defined manometric criteria for named esophageal motility disorders. We present a pictorial atlas of motility disorders. Achalasia types, esophagogastric junction obstruction, absent contractility, distal esophageal spasm, hypercontractile esophagus (jackhammer), ineffective esophageal motility, and fragmented peristalsis are depicted with high-resolution manometry plots.

## RESUMO

A manometria de alta resolução mudou vários paradigmas da motilidade digestiva. A Classificação de Chicago, na versão 3.0, definiu critérios manométricos para as doenças da motilidade esofagiana. O presente artigo é um atlas das dismotilidades descritas. Tipos de acalásia, obstrução ao nível da junção esofagogástrica, contrações ausentes, espasmo esofagiano distal, esôfago hipercontrátil, motilidade esofagiana ineficaz e peristalse fragmentada são mostradas em traçados de manometria de alta resolução.

## INTRODUCTION

**Figure 1:**
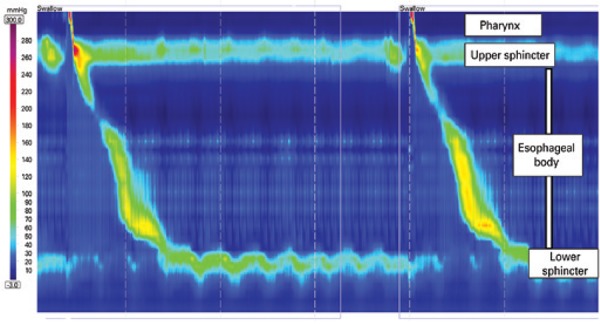
Normal high resolution manometry

High resolution manometry (Figure 1) has clear and inherent advantages over conventional manometry, despite its higher cost.^(1)^ High resolution manometry detailed analysis of esophageal peristalsis changed several esophageal motility paradigms, including new manometric parameters and different classification for named “motility disorders based on pressure topography”, the Chicago classification,^(2)^ which was recently revised.^(3)^

We present a pictorial atlas of the motility disorders according to the 3.0 Chicago Classification with high-resolution plots.

### Achalasia

**Figure 2:**
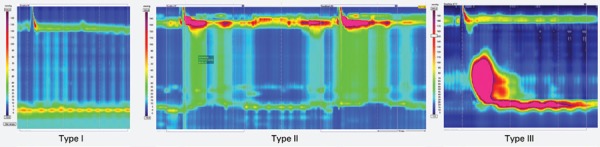
Achalasia types

Chicago Classification divided achalasia into three subtypes according to esophageal pressurization^(4)^ (Figure 2). Type I is characterized by 100% failed contractions and no esophageal pressurization; type II has panesophageal pressurization in at least 20% of swallows; and type III is defined by the presence of preserved fragments of distal peristalsis or premature contractions for at least 20% of the swallows.^(3)^ This classification may be applied to Chagas' disease esophagopathy as well, although type III is rarely, if ever, seem.^(5)^

### Esophagogastric junction obstruction

**Figure 3:**
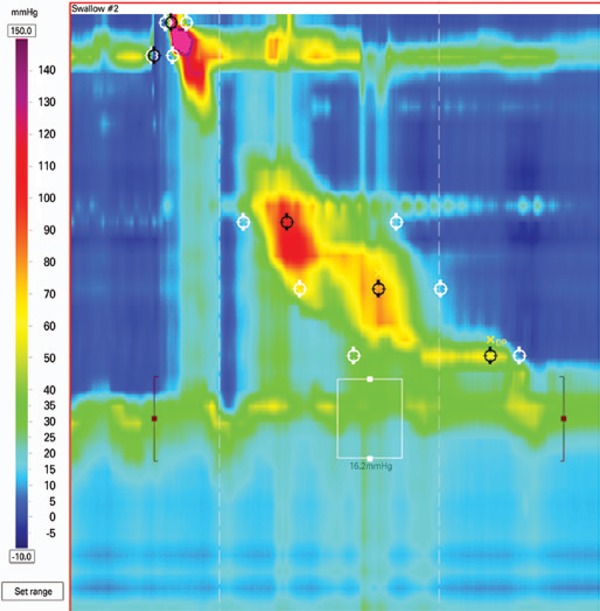
Esophagogastric junction obstruction in a patient after Nissen operation

Esophagogastric junction obstruction (Figure 3) is characterized by an elevated residual pressure of the lower esophageal sphincter (LES) measured by a new and more sophisticated tool, the integrated relaxation pressure^(6)^ in the absence of criteria for achalasia (absence of peristalsis).^(3)^ This parameter measures the mean pressure of the 4 seconds of maximal deglutitive relaxation in the 10-second window beginning at the beginning of the swallow (upper sphincter relaxation). It is a rare finding usually present in patients with dysphagia after operations at the esophagogastric junction.^(7)^

### Absent contractility

**Figure 4:**
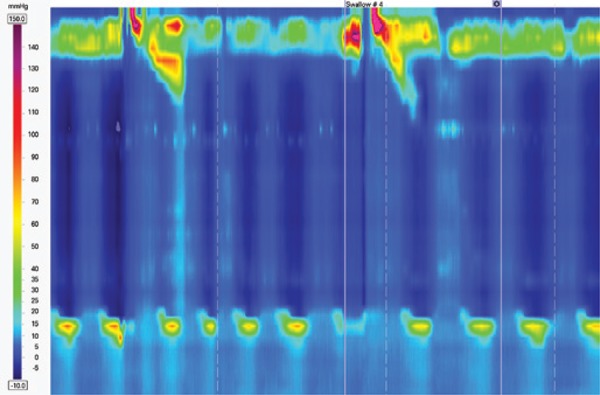
Absent contractility in a patient with scleroderma

Absent contractility is characterized by aperistalsis in the setting of normal LES relaxation and absence of esophageal pressurization^(3)^ (Figure 4). This finding may be noticed in patients with connective tissue diseases, end-stage gastroesophageal reflux disease etc.

### Distal esophageal spasm

**Figure 5:**
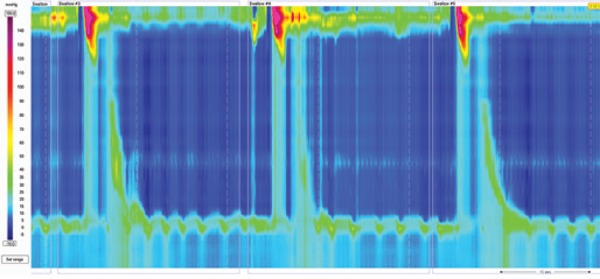
Distal esophageal spasm

Distal esophageal spasm is defined by over 20% of premature contractions as measured by a new parameter, the distal latency (DL) <4.5 seconds^(3)^ (Figure 5). The DL is the time interval between the beginning of the upper sphincter relaxation and the contractile deceleration point the manometric representation of the transition from the esophageal body to the epiphrenic ampulla regarded as an inflection of the peristaltic axis within 3cm of the proximal margin of the LES.^(8)^

### Hypercontractile esophagus

**Figure 6:**
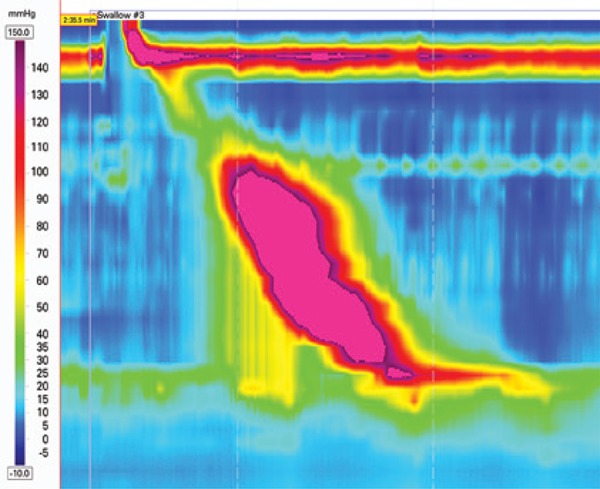
Hypercontractile esophagus

Hypercontractile esophagus (Jackhammer esophagus) is characterized by at least two swallows with hypercontractility as measured by the distal contractile integral (DCI)^(3)^ (Figure 6). The DCI measures the contractile vigor combining the amplitude *versus* duration *versus* length of the distal esophageal contraction exceeding 20mmHg from the transition zone to the proximal margin of the LES.^(9)^ Hypercontractility is defined by DCI >8,000mmHg.s.cm.^(3)^ It may occur with esophagogastric junction obstruction, gastroesophageal reflux disease and eosinophilic esophagitis.^(10)^

### Ineffective esophageal motility

**Figure 7:**
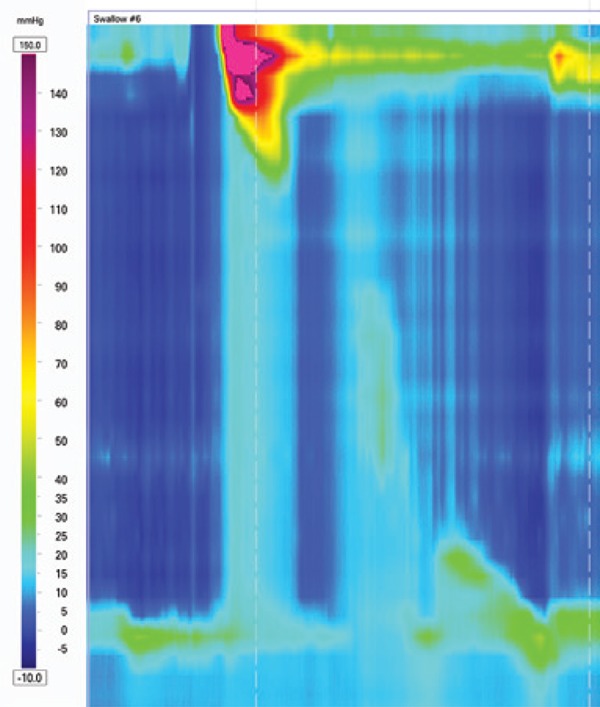
Ineffective esophageal motility in a patient with gastroesophageal reflux disease

Ineffective esophageal motility is defined by ≥50% ineffective swallows (failed or weak – DCI <450mmHg.s.cm)^(3)^ (Figure 7).

### Fragmented peristalsis

**Figure 8:**
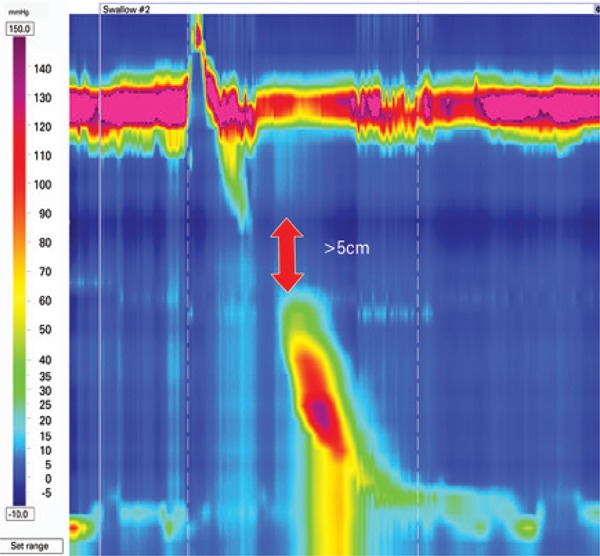
Fragmented peristalsis

Fragmented peristalsis ≥50% fragmented contractions with DCI >450mmHg.s.cm^(3)^ (Figure 8). Although patients with fragmented peristalsis are more prone to have dysphagia,^(11)^ its clinical significance is still elusive.

## DISCUSSION

Motility patterns to define named disorders have been motive of controversy since the era of conventional manometry. Different definitions exist although the classification by Richter was the most used by experts.^(12)^ High resolution manometry seems to bring a more intuitive and reproducible interpretation compared with conventional manometry,^(13)^ and more sophisticated tools to define old and new manometric parameters. Despite all improvements, and similarity with conventional manometry, some cases are still unclassified, and the real clinical significance of some Chicago Classification disorders is still under investigation.
